# A Bibliometric Analysis of Primary Aldosteronism Research From 2000 to 2020

**DOI:** 10.3389/fendo.2021.665912

**Published:** 2021-04-27

**Authors:** Chengyuan Wang, Hongwei Jing, Zuyu Sun, Jiaxi Yao, Xinyu Zhang, Tao Liu, Ying Wu

**Affiliations:** ^1^ Department of Urology, The First Hospital of China Medical University, Shenyang, China; ^2^ Department of Pulmonary Critical Care Medicine, The First Hospital of China Medical University, Shenyang, China; ^3^ Phase I Clinical Trails Center, The First Hospital of China Medical University, Shenyang, China

**Keywords:** primary aldosteronism, bibliometric analysis, VOSviewer, bibliographic item co-occurrence matrix builder, research hotspots

## Abstract

Thousands of papers on primary aldosteronism (PA) have been published in the last two decades. This study aimed to evaluate the research hotspots and future trends in PA research using bibliometric analysis. A total of 2,365 PA research papers between 2000 and 2020 were included. The dominant position of the United States in global PA research throughout this 20-year period was evident, and it was also the country most frequently involved in international cooperation. The University of Padua was the most productive institution and a leader in research collaboration. The Journal of Clinical Endocrinology & Metabolism was the most productive journal in terms of the number of publications on PA. Further, Mulatero P, Reincke M, Beuschlein F and Wu VC all made significant contributions to PA research. Five hotspots have been identified: (1) metabolic syndrome associated with PA; (2) molecular mechanisms of PA; (3) adrenal adenoma and adrenal cortex; (4) hypertension associated with PA; and (5) clinical monitoring parameters and diagnosis in patients with PA. Our results suggest that the molecular mechanisms of PA will remain research hotspots in the future. International collaboration is also expected to widen and deepen in the field of PA research.

## Introduction

Primary aldosteronism (PA), characterized by an increased aldosterone production, is the most frequent form of secondary hypertension (1, 2). PA patients have an increased risk of stroke, heart failure, coronary artery disease, atrial fibrillation (1, 3–5), renal damage (6, 7), diabetes (8, 9), metabolic syndrome (9, 10). PA is associated with reduced quality of life and an increased prevalence of mental fatigue, anxiety and depression (11–13). PA was first described by Jerome Conn in 1955 in a patient (14). During the past 65 years, especially in the last two decades, great progress has been made in the field of PA research regarding genetic and genomic mechanisms, pathophysiology, diagnostics, and therapeutics (2, 15, 16). However, there is still a lack of comprehensive reports that can assist researchers in obtaining an intuitive overview and reveal research trends in the PA research field.

Bibliometric analysis is a novel scientific method used to evaluate contributions to a research field, including those by countries, institutions, authors, and journals. Further, bibliometric analysis can predict the hotspots and trends within a certain research area through information visualization (17–19). However, few bibliometric studies have been performed in the field of PA research.

In the present study, we performed a comprehensive bibliometric analysis of PA research literature from 2000 to 2020, taking into account the number of annual publications, countries, international cooperation, institutions, journals, authors, and keyword co-occurrence visualization analysis. Furthermore, perspectives on progress in the field of PA research over the past two decades were considered. Overlay visualization maps of co-occurring keywords and double-clustering analysis were also performed in order to confirm the trends and hotspots in PA research. We hope that this study will provide new perspectives and a basis for future PA research.

## Materials and Methods

### Data Sources and Search Strategy

The Web of Science is one of the most influential databases of scientific literature. In this study, all data were retrieved from the Web of Science Core Collection (WoSCC) *via* the China Medical University library website. The retrieval strategy was TS = primary aldosteronism.

### Screening Criteria and Data Downloads

The publication period in the present study was limited to the period from 2000 to 2020. Non-English language, non-article, and non-review publications were excluded. WoSCC data including titles, author information, abstracts, keywords, journals, and references were downloaded in.txt format. To avoid the bias caused by frequent database updates, all literature retrieval and data downloads were completed on the same day (February 1, 2021). Two investigators (CW and JY) independently performed the search and had an agreement of 95% (kappa = (P0 − Pe)/(n − Pe) = 0.95 > 0.75), showing significant consistency (20).

### Statistical Analysis

In the present study, a comprehensive description of various publishing characteristics is provided, including authors, institutions, countries, journals, keywords, impact factor (IF), and Hirsch index (h-index). IFs were obtained from the 2019 Journal Citation Reports (JCR) to assess the scientific value of research (21). The value of the h-index was defined as the number of papers with citation number ≥ h and is considered to be an important indicator for assessing both the productivity and impact of the published work of scientists, journals, or countries (22). The filtered data from WoSCC was imported into the online analysis platform of literature metrology (http://bibliometric.com/) and VOSviewer 1.6.15 (Leiden University, Leiden, The Netherlands) for bibliometric analysis. Apache ECharts (https://echarts.apache.org/), a JavaScript-based data visualization tool, was used to visualize the annual number of publications and the number of cumulative publications in different countries/regions. The online bibliometric analysis platform was used to visualize international collaboration between countries. VOSviewer was used for analysis and visualization of bibliometric networks such as authors, institutions, journals, co-citations, and the keywords used in the articles (23). Network visualization maps and overlay visualization maps were generated using VOSviewer. The online bibliometric analysis platform and Microsoft Excel 2016 were used to assess the impact of authors, institutions, and journals. The filtered data from WoSCC were imported into Bibliographic Item Co-occurrence Matrix Builder (BICOMB) to construct a keyword-article binary matrix (24). The rows of the matrix represented publications, while the columns represented highly frequent keywords. Additionally, gCLUTO software 1.0 was used to perform double-clustering analysis, and to build mountain maps and heat maps based on the results of the clustering analysis (24).

## Results

### Trends and Annual Publications

**Figure 1:**
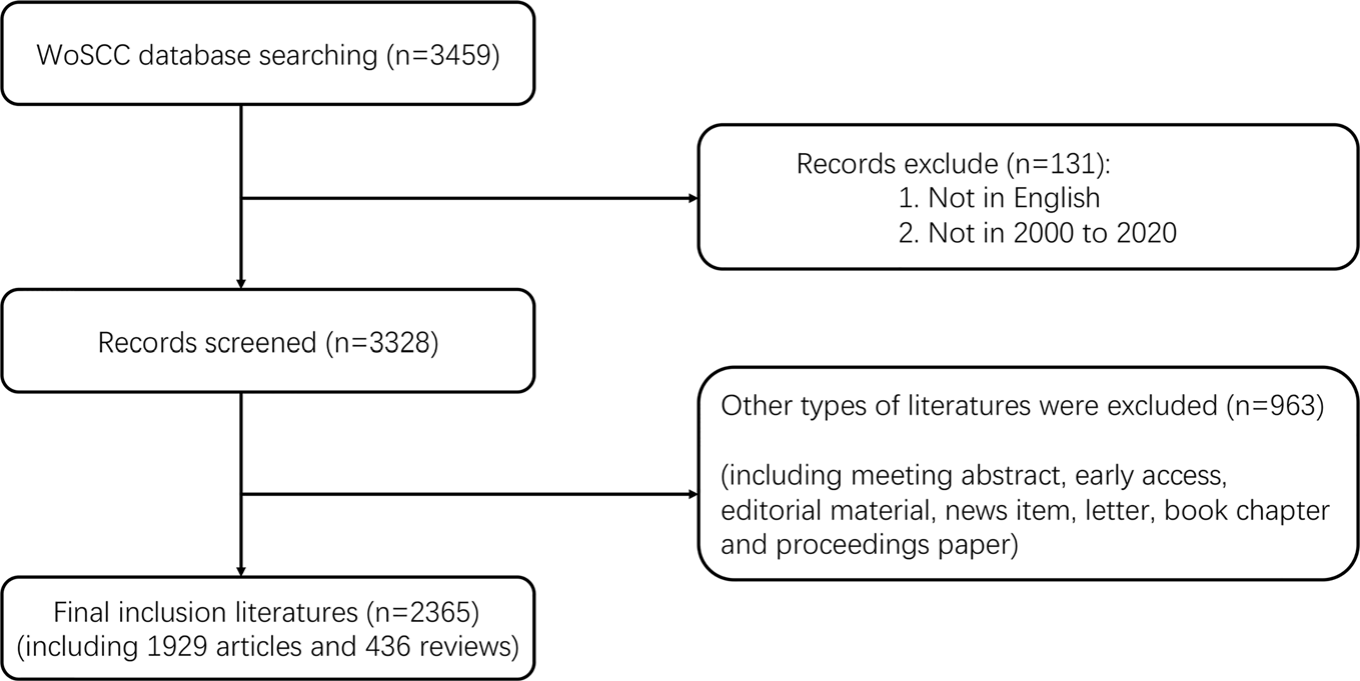
Flowchart of data filtration processing and excluding publications.

**Figure 2:**
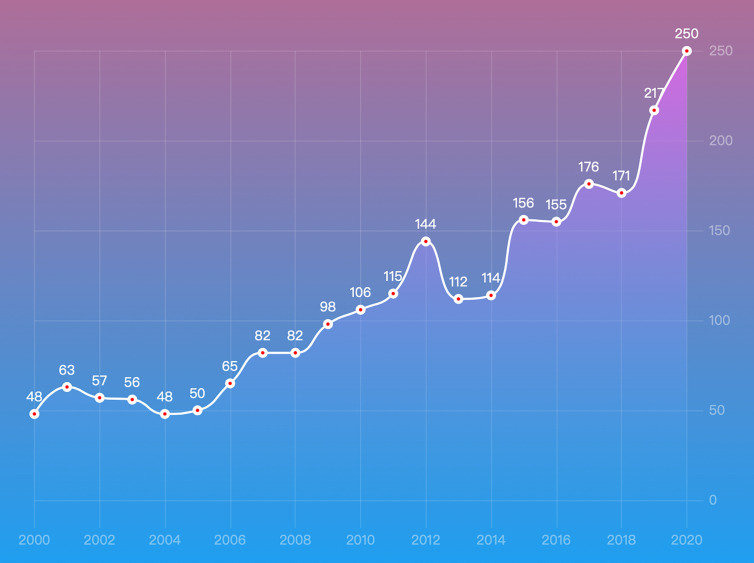
Annual number of the published publications in PA research from 2000 to 2020.

As shown in **Figure 1**, a total of 3,459 papers were identified, and 2,365 papers (1,929 articles and 436 reviews) from 2000 to 2020 were ultimately included according to the screening criteria. **Figure 2** shows the growth trend of the annual publications related to PA, from 48 in 2000 to 250 in 2020. Based on the WoSCC database, the 2,365 papers were cited 65,149 times, and each paper was cited an average of 27.55 times.

### Contribution of Countries and Institutions

**Figure 3:**
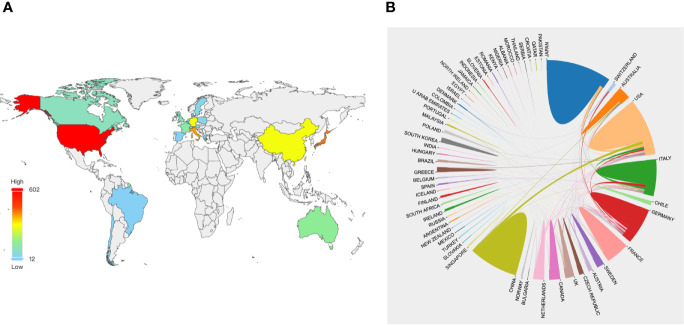
The distribution of countries or regions in PA research. **(A)** Distribution of PA literatures in the world map. The color of each country or region on the world map represents the amount of literature published, according to the color gradient in the lower left corner. **(B)** The network map of cooperation between countries or regions. Different colors represent different countries or regions, the area of each color represents the amount of literature published in each country or regions, and the thickness of the connecting line indicates the cooperation frequency.

According to the WoSCC database, 69 countries or regions contributed to publications on PA between 2000 and 2020. The top 24 countries or regions in terms of the number of publications (n > 10) on PA are presented on a world map in **Figure 3A**, and the top 10 are presented as numbers in **Table 1**. The United States was the largest contributor, with 602 papers published, followed by Japan (n = 419), Italy (n = 384), China (n = 293), Germany (n = 291), Australia (n = 151), France (n = 126), Netherlands (n = 100), Canada (n = 96), and England (n = 95). The United States and Japan contributed many more papers to the number of publications on PA than other countries or regions (**Figure 3A** and **Table 1**). Within the survey period, close cooperation between countries or regions around the world was extremely common. International cooperation analysis indicated that the United States was the country most frequently involved in international cooperation (**Figure 3B**).

**Table 1:** The top 10 countries or regions contributing to publications in PA research.

Rank	Country/Region	Records	Percentage. (N/2365), %
1	USA	602	25.455
2	Japan	419	17.717
3	Italy	384	16.237
4	China	293	12.389
5	Germany	291	12.304
6	Australia	151	6.385
7	France	126	5.328
8	Netherlands	100	4.228
9	Canada	96	4.059
10	England	95	4.017

**Figure 4:**
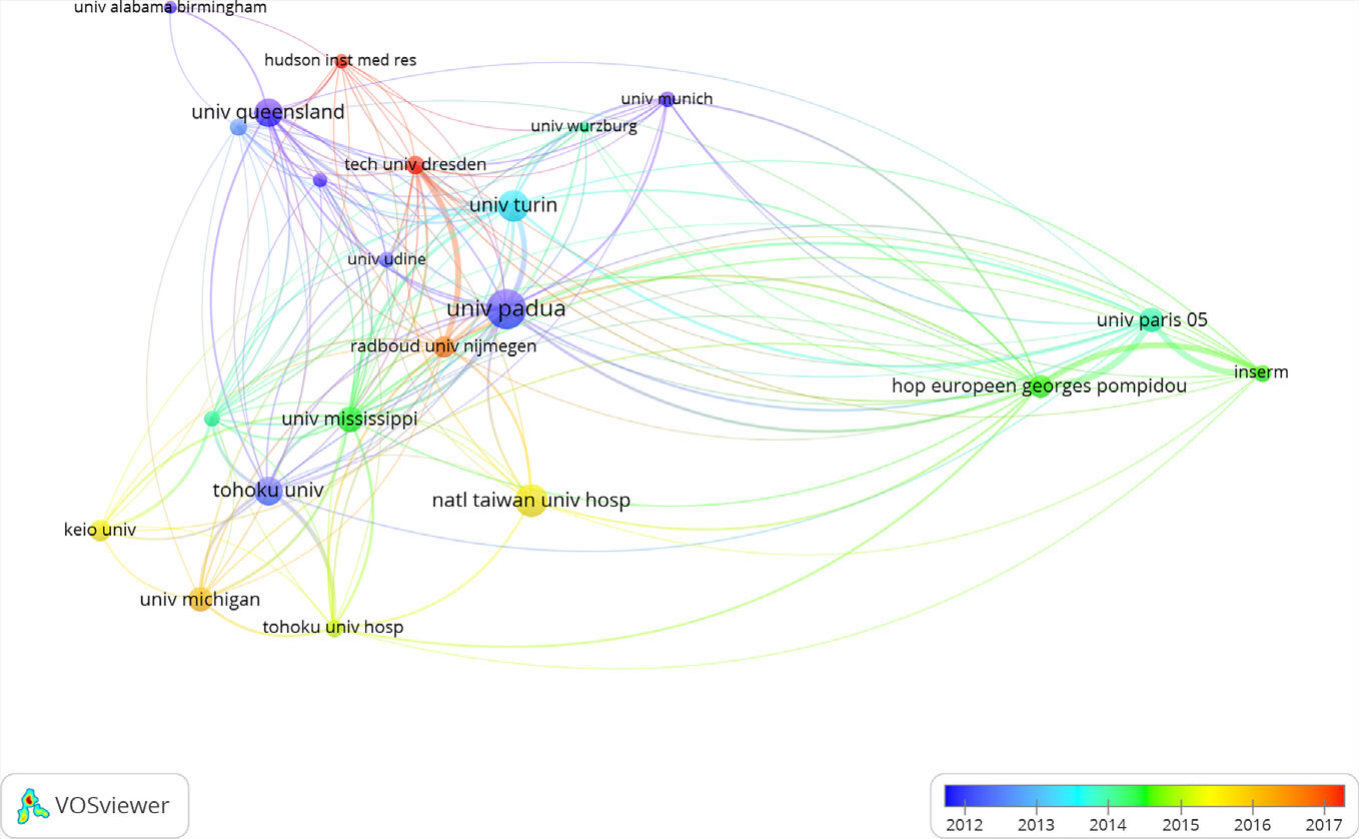
Co-authorship overlay visualization map of institutions. The color of each circle corresponds to the average publication year, the size of a circle is proportional to the number of literatures, and the thickness of the connecting line indicates the cooperation frequency.

The most productive institutions were also evaluated in our study. As shown in **Table 2**, with 140 papers published, the University of Padua was the most productive institution and was followed by National Taiwan University Hospital (n = 102), University of Turin (n = 96), Tohoku University (n = 86), University of Queensland (n = 82), University of Mississippi (n = 67), National Taiwan University (n = 63), University of Michigan (n = 63), University of Paris 5 (n = 63), and the Georges Pompidou European Hospital (n = 62). The collaboration network was generated using VOSviewer software, and the threshold was set to 20 as the minimum number of documents of an institution, while 1,000 was set as the minimum number of citations of an institution. Finally, 22 out of the 1,942 institutions were identified. During these two decades, University of Padua cooperated with almost all influential scientific institutions in studies on PA (**Figure 4**).

**Table 2:** The top 10 most productive institutions in PA research.

Rank	Country/Region	Records	Percentage. (N/2365), %
1	Univ Padua	140	5.920
2	Natl Taiwan Univ Hosp	102	4.313
3	Univ Turin	96	4.059
4	Tohoku Univ	86	3.636
5	Univ Queensland	82	3.467
6	Univ Mississippi	67	2.833
7	Natl Taiwan Univ	63	2.664
7	Univ Michigan	63	2.664
7	Univ Paris 05	63	2.664
10	Hop Europeen Georges Pompidou	62	2.622

### Contribution of Journals

In the present study, a comprehensive analysis of the contribution of journals with journal characteristics was provided, including journal titles, article counts, total citations, citations per article, IF (2019), quartile in category (2019), and h-index. The top 10 most productive journals in the field of PA research are listed in **Table 3**; in total, these journals published 809 papers, accounting for 34.21% of the total publications. *Journal of Clinical Endocrinology & Metabolism* (n = 165), *Hypertension* (n = 142), and *Journal of Hypertension* (n = 111) were the top three journals in terms of the number of publications on PA (**Table 3**). These three journals were the top three journals in terms of the highest total number of citations (6,399 *vs*. 4,394 *vs*. 2,146 citations, respectively), and they were also the top three journals with the highest average number of citations per paper (38.78 *vs*. 30.94 *vs*. 19.33 times, respectively). *Hypertension*, *Journal of Clinical Endocrinology & Metabolism*, and *European Journal of Endocrinology* had the highest IFs of any journals in 2019 (7.713 *vs*. 5.399 *vs*. 5.308, respectively). The highest h-index was 35, belonged to *Hypertension*. Among the top 10 most productive journals, *Journal of Clinical Endocrinology & Metabolism*, *Hypertension*, *Journal of Hypertension* and *European Journal of Endocrinology* were classified as Q1 according to the JCR 2019 standards (**Table 3**). The top 10 most highly cited publications are listed in **Table 4**.

**Table 3:** The top 10 most active journals that published articles in PA research.

Rank	Journal title	Article counts	Total number of citations	Average number of citations	IF(2019)	Quartile in category (2019)	H-index
1	Journal of Clinical Endocrinology & Metabolism	165	6,399	38.78	5.399	Q1	33
2	Hypertension	142	4,394	30.94	7.713	Q1	35
3	Journal of Hypertension	111	2,146	19.33	4.171	Q1	21
4	Hormone and Metabolic Research	88	940	10.68	2.562	Q3	14
5	European Journal of Endocrinology	66	997	15.11	5.308	Q1	23
6	Clinical Endocrinology	58	783	13.5	3.38	Q2	17
7	Journal of Human Hypertension	49	626	12.78	2.26	Q3	11
8	Hypertension Research	46	532	11.57	2.941	Q2	12
9	American Journal of Hypertension	45	696	15.47	2.669	Q3	13
10	Endocrine Journal	39	500	12.82	1.952	Q4	12

**Table 4:** The top 10 high-cited papers in PA research during 2000 to 2020.

Rank	Title	Authors	Year	Journal	Total citations
1	Case detection, diagnosis, and treatment of patients with primary aldosteronism: An endocrine society clinical practice guideline	Funder, John W. et al.	2008	Journal of Clinical Endocrinology & Metabolism	1073
2	Resistant hypertension: Diagnosis, evaluation, and treatment—A Scientific Statement from the American Heart Association Professional Education Committee of the Council for High Blood Pressure Research	Calhoun, David A. et al.	2008	Hypertension	998
3	Evidence for an increased rate of cardiovascular events in patients with primary aldosteronism	Milliez, P. et al.	2005	Journal of The American College Of Cardiology	960
4	A prospective study of the prevalence of primary aldosteronism in 1,125 hypertensive patients	Rossi, Gian Paolo. et al.	2006	Journal of The American College Of Cardiology	859
5	The Management of Primary Aldosteronism: Case Detection, Diagnosis, and Treatment: An Endocrine Society Clinical Practice Guideline	Funder, John W. et al.	2016	Journal of Clinical Endocrinology & Metabolism	777
6	A survey on adrenal incidentaloma in Italy	Mantero, F. et al.	2000	Journal of Clinical Endocrinology & Metabolism	636
7	Extensive personal experience—Increased diagnosis of primary aldosteronism, including surgically correctable forms, in centers from five continents	Mulatero, P. et al.	2004	Journal of Clinical Endocrinology & Metabolism	614
8	The incidentally discovered adrenal mass	Young, William F. et al.	2007	New England Journal of Medicine	602
9	Hyperaldosteronism among with resistant black and white subjects hypertension	Calhoun, DA. et al.	2002	Hypertension	445
10	Effect of spironolactone on blood pressure in subjects with resistant hypertension	Chapman, Neil. et al.	2007	Hypertension	428

### Contributions of Authors

**Figure 5:**
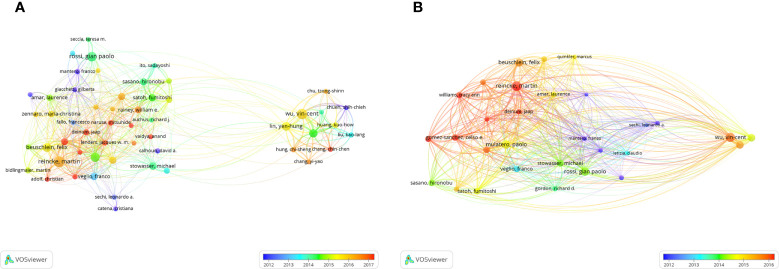
The distribution of authors in PA research. **(A)** Author co-authorship overlay visualization map. The color of each circle corresponds to the average publication year of the author, the size of a circle is proportional to the number of literatures published by the author, and the thickness of the connecting line indicates the cooperation frequency. **(B)** Author co-citation overlay visualization map. The color of each circle corresponds to the average publication year of the author, the size of a circle is proportional to the total number of citations of the author, and the thickness of the connecting line indicates the strength of the co-citation link.

The top 10 most productive authors in the field of PA research are presented in **Table 5**. Among them, Reincke M from the Ludwig-Maximilians-Universität München in Germany ranked first (n = 129). Mulatero P from the Department of Medical Sciences, University of Torino in Italy and Wu VC from the Department of Internal Medicine, National Taiwan University Hospital in Taiwan were the second most productive authors (n = 97). Furthermore, Stowasser M, Mulatero P, and Rossi GP were the top three authors with the highest total number of citations (3,885 *vs*. 3,421 *vs*. 3,392 times, respectively, **Table 5**). A co-authorship overlay visualization map was generated using VOSviewer software, and the threshold for the minimum number of documents by an author was set to 20. Finally, 55 authors who met the threshold were identified, and Mulatero P, Reincke M, Beuschlein F and Wu VC were shown to have cooperated closely (**Figure 5A**). A citation overlay visualization map was also generated using VOSviewer software, and the threshold for the minimum number of citations of an author was set to 1,000. Finally, 55 authors who met the threshold were identified, and it could be seen that Reincke M, Mulatero P and Wu VC had made significant contributions to the field of PA research (**Figure 5B** and **Table 5**).

**Table 5:** The top 10 most productive authors in PA research.

Rank	Author	Article counts	Total number of citations	Average number of citations	First author counts	First author citation counts	Corresponding author counts	Corresponding author citation counts
1	Reincke, M	129	3,241	25.12	4	261	65.25	40
2	Mulatero, P	97	3,421	35.27	26	1,614	62.08	45
3	Wu, VC	97	1,137	11.72	18	453	25.17	19
4	Rossi, GP	93	3,392	36.47	45	2,656	59.02	81
5	Beuschlein, F	87	2,251	25.87	8	173	21.63	18
6	Stowasser, M	76	3,885	51.12	24	858	35.75	51
7	Wu, KD	70	1,142	16.31	2	31	15.5	16
8	Lin, YH	70	890	12.71	7	148	21.14	20
9	Veglio, F	66	2,763	41.86	0	0	0	1
10	Williams, TA	65	1,673	25.74	12	402	33.5	18

### Analysis of Research Hotspots

**Figure 6:**
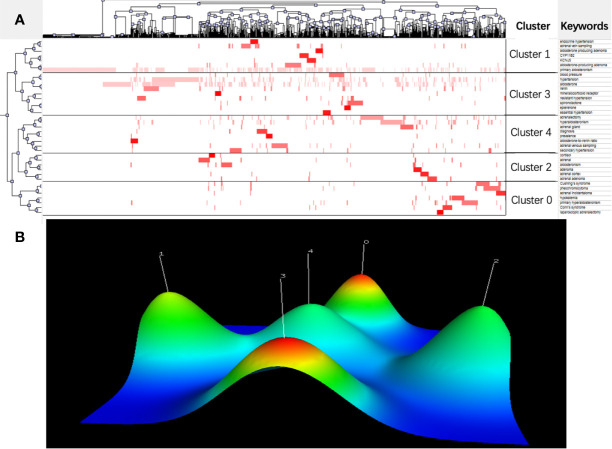
Research hotspots in the field of PA. **(A)** Visualized matrix of biclustering of highly frequent keywords in the research field of PA. Color of each blot represented the frequency of occurrence of keywords in all literatures. **(B)** Mountain visualization of biclustering of highly frequent keywords in the research field of PA. The height and color of the mountain are proportional to internal similarity and standard deviation of cluster (Blue: high deviation; Red: low deviation).

With an appearance of more than 20 times, 34 of the most frequent keywords were extracted from the included publications and are displayed in **Table 6**. Five clusters were sorted through double-clustering using gCLUTO. The relationship between publications and high-frequency keywords was visualized using a volcano map and matrix map (**Figure 6**). The matrix map is shown in **Figure 6A**, in which column labels represent articles, while row labels represent keywords. To combine similar rows in a single cluster, the rows of the initial matrix were reset and each cluster was partitioned by black horizontal lines. In the matrix map, the upper dendrogram represents article associations, while the left represents high-frequency keyword associations. The results of the volcano map in **Figure 6B** directly show the high-dimensional character of the data. In this three-dimensional image, five different mountains represent five different clusters, numbered from 0 to 4.

**Table 6:** Keywords of PA research hotspots.

Rank	Keywords	Frequency	Percentage (%)
1	primary aldosteronism	668	8.4653
2	aldosterone	421	5.3352
3	hypertension	356	4.5115
4	hyperaldosteronism	165	2.0910
5	adrenal vein sampling	146	1.8502
6	adrenalectomy	133	1.6855
7	aldosterone-producing adenoma	119	1.5080
8	renin	90	1.1405
9	aldosterone-producing adenoma	73	0.9251
10	primary hyperaldosteronism	71	0.8998
11	pheochromocytoma	66	0.8364
12	Cushing′s syndrome	63	0.7984
13	blood pressure	63	0.7984
14	secondary hypertension	59	0.7477
15	resistant hypertension	57	0.7223
16	adrenal gland	57	0.7223
17	spironolactone	55	0.6970
18	aldosteronism	51	0.6463
19	hypokalemia	46	0.5829
20	adrenal	45	0.5703
21	adrenal adenoma	38	0.4816
22	diagnosis	37	0.4689
23	Conn′s syndrome	36	0.4562
24	adrenal incidentaloma	31	0.3929
25	CYP11B2	29	0.3675
26	adrenal cortex	28	0.3548
27	KCNJ5	28	0.3548
28	adenoma	25	0.3168
29	essential hypertension	24	0.3041
30	endocrine hypertension	23	0.2915
31	prevalence	22	0.2788
32	eplerenone	22	0.2788
33	cortisol	21	0.2661
34	mineralocorticoid receptor	20	0.2535

The above 34 high-frequency keywords were divided into five clusters. All representative articles involved in each cluster were mined to further summarize hotspots in the field of PA Finally, five hotspots were identified using BICOMB and gCLUTO software packages:

Cluster 0: Metabolic syndrome associated with PA.
Cluster 1: Molecular mechanisms of PA.
Cluster 2: Adrenal adenoma and adrenal cortex.
Cluster 3: Hypertension associated with PA.
Cluster 4: Clinical monitoring parameters and diagnosis in patients with PA.

**Figure 7:**
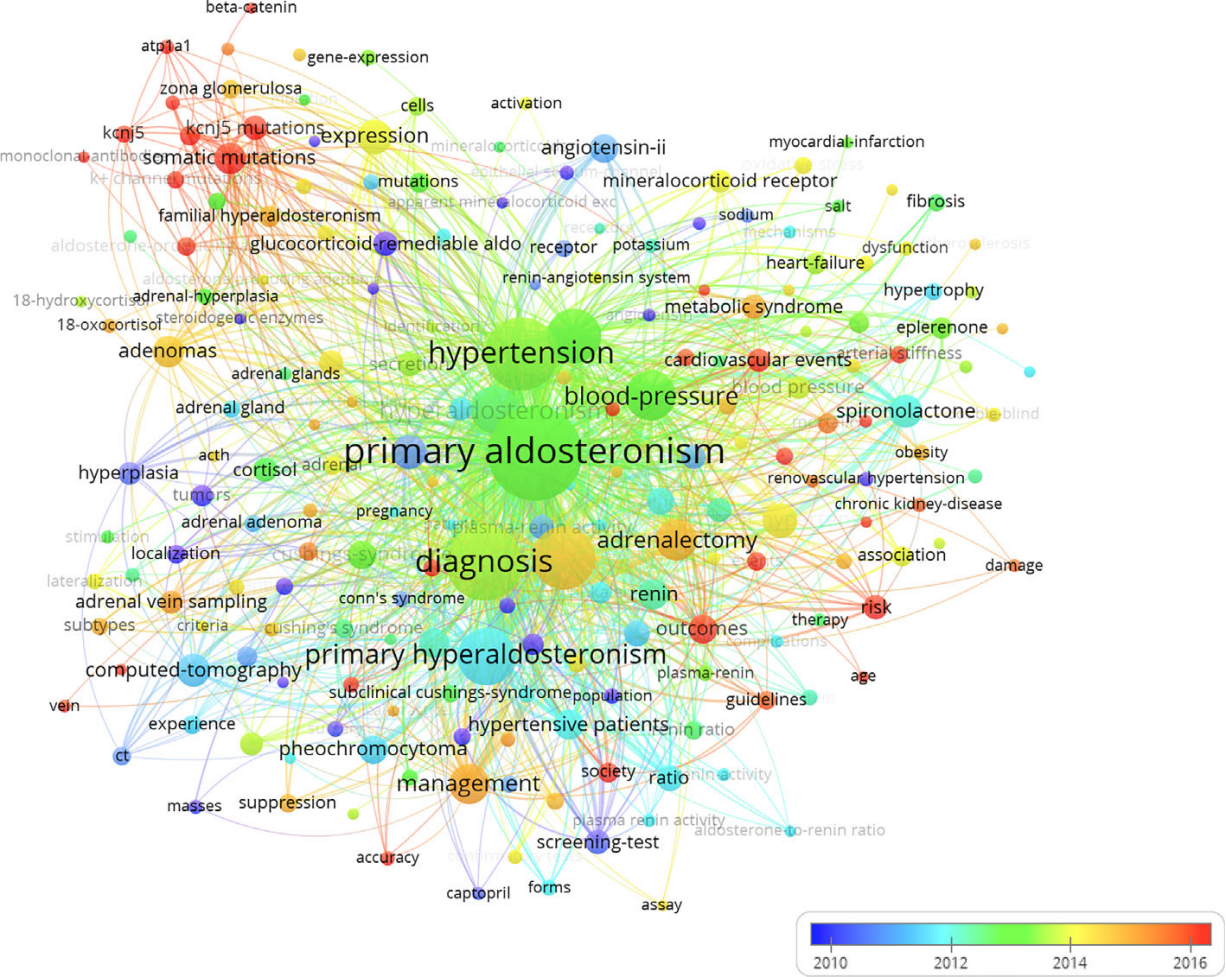
Keywords co-occurrence overlay visualization map. The color of each circle corresponds to the average publication year. The size of a circle is proportional to the frequency of occurrence of the keyword, and the thickness of the connecting line indicates the strength of the keywords co-occurrence link.

To explore the changes of hotspots over a period of time, a network visualization map of keyword co-occurrence was generated using VOSviewer software, and the results showed that the keywords “KCNJ5”, “K(+) channel mutations”, “somatic mutations”, and “KCNJ5 mutations” began to appear in the last 5 years (**Figure 7**).

## Discussion

In the era of the information explosion, bibliometric analysis can help scientific researchers to manage their knowledge and visualize knowledge structures more intuitively. By presenting visual results, bibliometric analysis can help new researchers in a specific field to grasp the overall trends in the field being investigated. It can also reveal milestone manuscripts, the most productive authors and institutions, and current research hotspots, as well as future trends (25–27). In our study, a comprehensive bibliometric analysis of global scientific publications in the field of PA research from 2000 to 2020 was performed.

The number of publications in a particular research field can reflect the productivity and developments in the field over time (28). In the present study, a total of 2,365 publications, including 250 literature in 2020, were included (**Figures 1**, **2**). The results showed that the number of publications in the field of PA was maintained at a substantial level in the two decades from 2000 to 2020, which suggests that PA remains a hot research field, and more and more scholars may participate in PA research.

The number of publications in a research field is an important indicator for evaluating the scientific research level of a country or institution (25, 28, 29). Our study showed that the United States and Japan were the two largest contributors to the number of publications on PA (**Figure 3A** and **Table 1**), highlighting their impact in the PA research field. The value of international collaboration in supporting innovation and addressing unmet challenges is well recognized worldwide (30). From 2000 to 2020, many countries or regions around the world collaborated on studies in the research field of PA. Furthermore, our results demonstrated that the United States had the highest collaboration performance, especially with China and Japan (**Figure 3B**). Meanwhile, University of Padua was identified as the most productive institution during the 20-year period (**Table 2**) and cooperated with almost all influential scientific institutions in the PA research field, including National Taiwan University Hospital, University of Turin, Tohoku University, and University of Queensland (**Figure 4**). These results showed that highly collaborative countries or institutions generally had a high academic level, suggesting that international cooperation will remain a future trend in the field of PA research.

Journal indicators obtained from bibliometric analysis can provide a reliable reference for researchers to search documents or submit manuscripts (31, 32). All the top 10 journals publishing literature on PA were included in the category of “Internal medicine” or “Endocrinology”. Our results showed that *Journal of Clinical Endocrinology & Metabolism* published the highest number of PA-related papers and had the highest number of total citations (**Table 3**). Our results also showed that the most frequently cited publication was a clinical practice guideline written by John W Funder and colleagues from Prince Henry’s Institute of Medical Research in Australia, and that “Case detection, diagnosis, and treatment of patients with primary aldosteronism: An endocrine society clinical practice guideline”, was a milestone in the PA research field in the two decades from 2000 to 2020 (33) (**Table 4**). These results suggest that these active journals and highly cited papers can provide a reliable reference for scholars concerned with the progress of PA research, and PA plays an important role in the fields of endocrinology and internal medicine.

Based on the WoSCC database, Reincke M published the highest number of PA-related papers, while Stowasser M had the highest number of total citations (**Table 5**). Furthermore, Mulatero P, Reincke M, Beuschlein F and Wu VC cooperated closely and published a considerable number of highly cited publications, as evidenced in the co-authorship overlay network visualization map and citation overlay visualization map (**Figure 5A**, **B**). Therefore, they can be regarded as the leaders in the PA research field.

Because of the heterogeneity of the PA research field, we divided the keywords in our study into five clusters *via* double-clustering analysis (**Figure 6**). Cluster 0 is related to PA-related metabolic syndrome. The abnormal glucose metabolism caused by insulin resistance is related to the excessive production of aldosterone, which is the main cause of metabolic dysfunction in patients with PA (34). Cluster 1 is related to molecular mechanisms of PA. In recent years, somatic mutations are identified in genes associated with PA, KCNJ5, CACNA1D, ATP1A1 and ATP2B3 (35–37). In general, the emergence of Next-generation sequencing (NGS) technology has driven researchers to understand the pathogenic and molecular mechanisms of PA (38). Cluster 2 is related to adrenal adenoma and adrenal cortex. PA results from excessive production of aldosterone by the adrenal cortex. Adrenal adenoma is considered a benign neoplasm of the adrenal cortex. In addition, genetic studies have helped to understand the relationship between benign aldosterone-producing adrenocortical proliferation and ion channel mutations (2, 39, 40). Cluster 3 is related to hypertension associated with PA. PA accounts for 5–10% of all hypertension patients and exist in 20% of those with resistant hypertension. Our understanding of PA-related hypertension has increased tremendously during the last two decades and exploring how PA leads to hypertension is the key to improve the outcome of long-term diseases and improve the quality of life of PA patients (2, 6, 41). Cluster 4 is related to clinical monitoring parameters and diagnosis in PA patients. Adrenal vein sampling (AVS), the most recommended procedure for lateralization in PA, has many limitations such as required technical expertise, increased costs, and potential complications (42). As a consequence, the development of new non-invasive imaging techniques and monitoring parameters is conducive to timely diagnosis, provides appropriate treatment, and prevents deleterious cardiovascular outcomes of PA, which is also a challenge for doctors and researchers in the future (2, 43).

Keyword co-occurrence network visualization analysis is a widely accepted method for determining research hotspots and predicting research trends (44). Our results indicated that the keywords such as “KCNJ5”, “K(+) channel mutations”, “somatic mutations”, and “KCNJ5 mutations” appeared frequently in the last 5 years, suggesting that the study of the genomics and mechanisms of PA will remain research hotspots over the next few years. Multicenter studies have reported that the most frequent genetic abnormalities are KCNJ5 somatic mutations, which were found in approximately 40% of aldosterone-producing adenoma (APA), a subtype of primary aldosteronism (35–37). Somatic mutations in KCNJ5, which encodes the G­protein-coupled inward rectifier K+ channel, have been considered as a cause of PA (37, 40). Our analysis also suggests that scientists are still trying to gain a comprehensive understanding of PA, and we expect scientists to make breakthroughs in the pathogenesis and management of this condition in the near future.

However, there were some limitations in our study. Firstly, the WoSCC database is updated continuously and dynamically. Therefore, our results are temporary in nature. Secondly, non-English publications were excluded. Hence, a discrepancy may exist between our results and the real publication characteristics.

In conclusion, the annual number of publications on PA grew in the two decades between 2000 and 2020. The United States was the leading country in this research field, while the University of Padua also achieved important research results and played a certain role in promoting the development of PA research. Furthermore, Mulatero P, Reincke M, Beuschlein F and Wu VC made significant contributions to this research field. Research hotspot analyses suggest that the molecular mechanisms of PA will remain research hotspots in the future. International collaboration was also prevalent, and it is expected to widen and deepen in the future. These results provide new perspectives for the study of PA and may have a beneficial effect on further study regarding the etiology, diagnosis, and treatment of this condition.

## Data Availability Statement

The raw data supporting the conclusions of this article will be made available by the authors, without undue reservation.

## Author Contributions

YW and TL conceived the study. CW, HJ, ZS, JY, and XZ participated in statistical analysis. CW wrote the manuscript. All authors contributed to the article and approved the submitted version.

## Funding

The study received funding from The Education Department of Liaoning Province (FWZR2020003).

## Conflict of Interest

The authors declare that the research was conducted in the absence of any commercial or financial relationships that could be construed as a potential conflict of interest.

## Abbreviations

PA, Primary aldosteronism; WoSCC, Web of Science Core Collection; BICOMB, Bibliographic Item Co-Occurrence Matrix Builder; IF, impact factor; H-index, Hirsch index; JCR, Journal Citation Reports; AVS, Adrenal vein sampling.
